# Ultra-fast polymer optical fibre Bragg grating inscription for medical devices

**DOI:** 10.1038/lsa.2017.161

**Published:** 2018-03-23

**Authors:** Julien Bonefacino, Hwa-Yaw Tam, Tom S Glen, Xin Cheng, Chi-Fung Jeff Pun, Jian Wang, Po-Heng Lee, Ming-Leung Vincent Tse, Steven T Boles

**Affiliations:** 1 Photonics Research Centre, Department of Electrical Engineering, The Hong Kong Polytechnic University, Hung Hom, Kowloon, Hong Kong, China; 2 Division of Urban Environment, Department of Civil and Environmental Engineering, The Hong Kong Polytechnic University, Hung Hom, Kowloon, Hong Kong, China

**Keywords:** bio-sensing, fibre Bragg grating, photosensitive dopant, polymer optical fibre

## Abstract

We report the extraordinary result of rapid fibre Bragg grating inscription in doped polymer optical fibres based on polymethyl methacrylate in only 7 ms, which is two orders of magnitude faster than the inscription times previously reported. This was achieved using a new dopant material, diphenyl disulphide, which was found to enable a fast, positive refractive index change using a low ultraviolet dose. These changes were investigated and found to arise from photodissociation of the diphenyl disulphide molecule and subsequent molecular reorganization. We demonstrate that gratings inscribed in these fibres can exhibit at least a 15 times higher sensitivity than silica glass fibre, despite their quick inscription times. As a demonstration of the sensitivity, we selected a highly stringent situation, namely, the monitoring of a human heartbeat and respiratory functions. These findings could permit the inscription of fibre Bragg gratings during the fibre drawing process for mass production, allowing cost-effective, single-use, *in vivo* sensors among other potential uses.

## Introduction

Fibre Bragg gratings (FBGs) can be easily inscribed in silica optical fibres in <1 min, but the grating inscription time in polymethyl methacrylate (PMMA) optical fibres is much longer, and in some polymer optical fibres (POFs), this process takes >1 h^1, 2^. To date, the shortest reported inscription time using a 325 nm He-Cd laser is 1 s^3, 4^, while a much faster grating inscription of 15 ns (1 pulse) was recently reported using an expensive high peak-power 248 nm excimer laser^5^. These times are too slow for the grating inscription to be carried out during the fibre-drawing process at a reasonable cost. Furthermore, PMMA starts to degrade upon irradiation with ultraviolet (UV) light below ~300 nm^6^. Historically, PMMA was the preferred material for producing POF^7, 8, 9^ because of its ease of fabrication, low cost, high optical transparency in the visible region^10^ and refractive index similar to that of a conventional silica telecommunication optical fibre^7, 11^ (1.457 @643 nm for silica and 1.488 @632 nm for PMMA). Here we demonstrate the fabrication of a single-mode POF using a unique core dopant, diphenyl disulphide (DPDS), which is responsible for both the increase in the core refractive index and the photosensitivity. We record the fabrication of FBGs that are UV-inscribed in 7 ms, which is over 140 times faster than previous results reported for PMMA fibres^3, 4^, and we report the experimental results of a direct comparison of silica and polymer FBGs for monitoring vital signs.

Furthermore, we investigate the mechanism for this effect using Raman spectroscopy and chemical modelling.

POF sensors based on PMMA are a promising sensing technology, especially for the biomedical industry. PMMA fibres are biocompatible and immune from magnetic interference; they do not produce shards when they break, and they are inexpensive to produce^12, 13^.

An interesting development currently being investigated is the use of a single conventional silica FBG sensor attached to the wrist to measure multiple vital signs of subjects^14^, including heart rate, blood pressure, body temperature, respiratory status and level of consciousness. The main issue in this application is that silica FBGs break easily and can produce sharp shards. To our knowledge, there are no reported results using POF-FBG for heartbeat measurements. The current state of the art shows that different techniques are employed^15, 16, 17^, which require many pieces of expensive equipment. Another potential application of FBG sensors is in minimally invasive surgery. However, the Young’s modulus of silica (73 GPa^18^) is much higher than that of surgical tools made of super-elastic nitinol (41 GPa^19^), and small-diameter silica fibres must be used to reduce stiffness for better sensitivity^20^. PMMA typically has a much smaller Young’s modulus (~4 GPa^21^), which makes it more suited for the aforementioned applications, and it is more sensitive than silica FBG for strain measurements. This work is a crucial step towards the development of a body-safe, sensitive, reliable and disposable fibre optics multi-vital-signs sensing system. Bio-sensing is one example application, but these results could also be of interest to the wider precision sensor industry and have potential in transportation, Internet of Things and automation applications.

## Materials and methods

### Polymer optical fibre fabrication

All of the fibres presented in this article were drawn from preforms that were fabricated using the ‘pull-through’ method with a core preform inserted in a cladding preform. This method, although similar, is different from the Teflon string method^12, 22, 23^, in which the core monomers are poured directly into a cladding preform. Details about preform fabrication using the ‘pull-through’ method are given in Supplementary Information. The fabricated fibres were 120 μm in diameter, with a core of 5.5 μm.

### Fibre Bragg grating inscription

A KIMMON He-Cd 325 nm laser (IK3501R-G) was used to fabricate the FBGs. Four mirrors were employed, which directed the beam through a 150-mm plano-convex cylindrical lens that focussed the beam onto the fibre. The fibre was secured on a Newport 125-μm V-groove using tape. Two layers of tape were adhered on each side of the V-groove, and a phase mask (Ibsen) was placed on top of the tape. The tape thickness is approximately 120 μm, which allows a small gap between the fibre and the phase mask; it prevents any particle transfer from the fibre to the phase mask. The pitch of the phase mask was 1046.3 nm. Prior to FBG fabrication, the fibre was taped onto a hot plate for 1 min and was cut using a hot blade^24^.

A beam shutter (Thorlabs SH05) was placed in the optical path and controlled by a shutter controller (Thorlabs SC10), which allows a minimum irradiation time of 7 ms. To expand the beam, a beam expander (Thorlabs BE10-UVB) was mounted between the third and fourth mirrors, forming a 12-mm-long elliptical beam on the V-groove. All of the FBGs presented in this manuscript are strictly 10 mm long, delimited by tape. The optical power of the UV beam measured after the lens was ~25.5 mW (using Thorlabs S120VC). The same optical output power was used to inscribe all of the FBGs presented in this work. This inscription method offers two main advantages: (1) it shortens the FBG inscription time, and (2) it can irradiate the whole phase mask at once. This irradiation scheme required only low power density (1.53 mJ cm^−2^) and was sufficient for writing good-quality FBGs in just 7 ms. All of the fibres used in the manuscript were annealed at 80 °C for 2 days prior to grating inscription to remove the stress induced during the drawing process. Details concerning the FBG fabrication process and interrogation of the sensor are given in Supplementary Information.

### Filtering protocol to extract the heartbeat and respiration signals

To extract meaningful heartbeat and respiration signals, a Labview program was used to filter the raw data collected by the FBG sensor. An adjustable bandpass filter was applied to remove noise signals. For the respiration measurement, low- and high-cut frequencies of 0.15 and 0.3 Hz were applied, respectively; for the heartbeat measurement, 2 and 8 Hz were applied, respectively. Automatic counting of peak-and-trough of the resultant signal was performed by setting different thresholds of the wavelength shift for different subjects.

### Raman spectroscopy

Fabricated POF and preforms were cut into ~2 cm lengths and were taped together using Kapton tape and held vertically inside a cylindrical mould. The mould was filled using Buehler’s EpoKwick® epoxy resin and left to set overnight. The embedded fibres were removed from the mould and cut into three sections. These sections were ground and polished. Silicon carbide waterproof paper of 150 grit was used to remove the rough edges from the cutting and to ensure that the sections were of equal height. They were then attached to a plate for automatic milling. Sequential grits of up to 2000 were used on an EQ-Unipol-802 machine to obtain a smooth top surface. This step was followed by alumina slurry and polishing pads, with particle sizes of 3, 0.3 and 0.05 microns sequentially, giving a mirror finish. The sections were rinsed with deionized water before sonication in deionized water for 15 min to ensure a clean surface. Once dry, POFs could be imaged using a Leica DM4000 optical microscope to ensure that the core region was visible.

Raman spectra of the POF cross-sections were taken using a Horiba LabRAM HR 800 spectrometer, with a 633 nm laser. Spectra with good signal-to-noise ratios were acquired between Raman shifts of 200 and 3500 cm^−1^ using a dwell time of 6 s, averaged 3 times, with subpixel averaging of 4. Faster Raman spectra were also acquired using a dwell time of 3 s, zero averaging and no subpixel averaging. Such spectra were then taken at several different locations and averaged, producing very similar graphs to the longer dwell time acquisitions. This procedure was performed to ensure that there was no spatial variation across the sample.

### Chemical modelling

Chemical modelling was accomplished by performing *ab initio* electronic structure calculations using the Gaussian 09 program package^25^. Structural optimizations were obtained using the B3LYP hybrid density functional with a 6–311++G(d,p) basis set. Frequency calculations at the same level were performed to investigate whether the obtained stationary point is an isomer. The reported relative Gibbs free energy (Δ*G*) and enthalpy (Δ*H*) are calculated at 298 K, and the relative electronic energy (Δ*E*) is corrected by including a zero-point energy (ZPE).

## Results and discussion

### Fibre characteristics

Typically, the photosensitivity and refractive index of the core of a POF are increased by using two different materials. For example, dopants such as *trans*-4-stilbenemethanol were used to enhance the photosensitivity and benzyl methacrylate^12, 26^ or diphenyl sulphide^3^ to increase the refractive index. In some POFs, benzyl methacrylate was replaced by diphenyl sulphide because of the lower transmission loss. Here a single active chemical compound, DPDS, was used to provide for both the ultra-photosensitivity and the increase of the refractive index in the fibre core, albeit with a slightly higher loss compared with pure PMMA, without consequence for the intended application. Preforms were fabricated using the ‘pull-through’ technique described in Materials and Methods and further detailed in Supplementary Information, which allows control of the refractive index profile, an essential condition for the fabrication of a single-mode fibre. The cladding was made of pure PMMA, and the core consisted of PMMA_92%wt_+ DPDS_8%wt_. Note that the dopant is sometimes referred to in %mol of MMA monomer. In the present case, the core dopant concentration was 4%mol.

The average transmission loss of the DPDS POF was measured by the multiple cutback method. The fibre was cut by hand using a hot plate and razor blade^24^. A SLED source (EXALOS EXS210018-01) and a fibre optic power meter (Thorlabs PM320E) with an integrating cavity detector head (Thorlabs S142C) were used for the measurements. The fibre has loss values of 87.12 dB m^−1^ at 1550 nm and 26.67 dB m^−1^ at 870 nm for a concentration of 8%wt (4%mol) of DPDS in the fibre core. The high attenuation value is mainly attributed to the current laboratory conditions and the simple chemical purification process and can be significantly reduced by fabricating the preform in an enclosed environment^27^. The attenuations of commercial PMMA fibres at the wavelengths of 870 and 650 nm are ~5 and ~0.5 dB m^−1^ ^10^, respectively. We measured the additional loss induced by DPDS at 0%wt, 2%wt (1%mol), 4%wt (2%mol) and 8%wt (4%mol) (described in Supplementary Information), and we obtained 0.98 and 1.75 dB m^−1^%wt^−1^ at 650 and 870 nm, respectively. This finding indicates that DPDS-doped PMMA-POF with a loss of ~2.5 dB m^−1^ at 650 nm can be fabricated, which permits sensor interrogation of over 2 m with a round-trip loss of approximately 10 dB.

### Fibre Bragg grating inscription set-up

**Figure 1:**
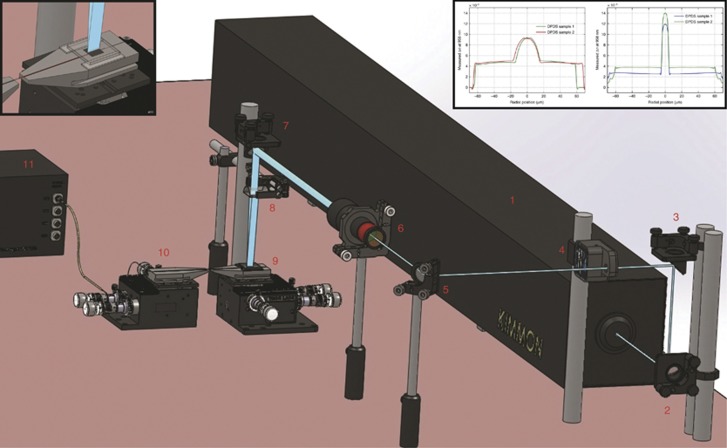
Polymer optical FBG fabrication setup using a static beam. The setup used 4 mirrors (2, 3, 5 and 7) to guide the laser beam from (1) to (9). The beam shutter (4) is inserted in the optical path after the second mirror (3), and the 10 × beam expander (6) was placed after the third mirror (5) to expand the beam. The plano-convex cylindrical lens (8) focuses the beam onto the fibre. The FBGs' spectra was recorded with an FBG interrogator (11) via an angled-cleaved single mode fibre (10). The upper left corner shows the POF with the phase mask placed on top of the V-groove. The measured refractive index profiles at 958 nm of two samples of DPDS core-doped POFs fabricated using the Teflon string method (left) and DPDS core-doped fibres fabricated using the ‘pull-through’ method (right) are shown in the upper right corner.

The optical set-up for the millisecond grating inscription is depicted in Figure 1. The single-core dopant is sufficient to increase the refractive index and to substantially enhance the photosensitivity to permit the inscription of FBGs in milliseconds. Moreover, the POF operates in the single-mode regime with a good refractive index profile (see inset of Figure 1).

The refractive index profiles of POFs that were fabricated using two different methods were measured with a fibre index profiler (IFA-100™ from Interfiber Analysis®) in Sharon, MA, USA. Examples of the measurements for two fibres with a larger core are shown in the left-hand side in the inset of Figure 1. The profiles for fibres fabricated using the Teflon string method^12^ (left) show significant dopant diffusion from core to cladding. The diffusion occurred during the core polymerization process, which makes it difficult to produce single-mode fibres. The results for fibres fabricated using the ‘pull-through’ technique show virtually no dopant diffusion (right); the measured profiles show an excellent abrupt change in the refractive index between the core and the cladding.

### Fabrication of ultrafast FBGs

**Figure 2:**
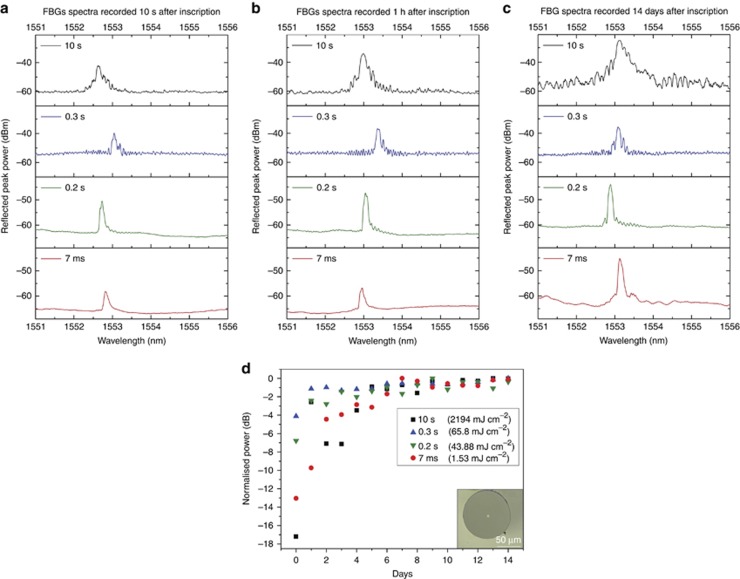
Measured reflection spectra of FBGs inscribed with different UV irradiation times in the POF and their growth post-irradiation. (**a**) Gratings recorded 10 s after inscription, (**b**) 1 h after inscription and (**c**) 2 weeks after inscription. (**d**) Growth of the normalized reflected peak power of FBGs written in 10 s, 0.3 s, 0.2 s and 7 ms within 2 weeks post-irradiation. The inset shows the end face of the fabricated single-mode POF.

The novel core dopant and irradiation scheme required only low power density (1.53 mJ cm^−2^) and was deemed to be sufficient for writing FBG in just 7 ms. Figure 2a–2c shows the spectra recorded 10 s, 1 h and 14 days after UV irradiation for FBGs written in 10 s, 0.3 s, 0.2 s and 7 ms. Note that all of the FBGs appeared almost immediately after the UV irradiation. The most important result is the formation of the millisecond FBGs. As shown in Figure 2a, 7 ms FBGs exhibited signal-to-noise ratios of 7 dB, 10 s after UV irradiation. Figure 2b and 2c, shows that all of the FBGs experienced significant post-irradiation growth. At 14 days after UV irradiation, the signal-to-noise ratio of the 7 ms FBGs increased to 14.8 dB.

The FBGs written with 0.3 s or longer UV irradiation time exhibited a higher noise level and larger full-width-half-maximum than FBGs inscribed in a shorter time. The full-width-half-maximum of FBGs inscribed in less than 0.3 s was measured to be 80 pm, 14 days after the inscription. Millisecond FBGs exhibited suppressed side lobes with a high signal-to-noise ratio, which is desirable for sensing applications. These breakthrough results demonstrate the feasibility of writing FBGs in POFs during the fibre-drawing process.

### Stability of the gratings

Gratings written in polymer fibres can be unstable^28^, and their peak wavelength and power can fluctuate with time. One reason for the instability is the stress induced in the POFs during the fibre-drawing process^29^. In practice, the stress is slowly released by thermal annealing at ~80 °C for many hours^30^. Figure 2d shows the normalized reflected peak power fluctuations for gratings left in an air-conditioned laboratory without strict control of the temperature or humidity for up to 2 weeks post-irradiation. Significant fluctuations in the peak power were observed during the first few days after inscription. All of the FBGs stabilized after approximately a week, and the growth of the reflected peak power in milliseconds FBGs is more pronounced for the 7-ms grating. It is apparent from Figure 2a–2c that there is a common growth regime for gratings written with UV fluence below ~65 mJ cm^−2^ (0.3 s) because the peak stabilized at the same level. In general, the reflected peak power is proportional to the UV fluence up to a certain threshold, at which degradation occurs^31^. Interestingly, the growth of the 7-ms grating continued for months at a very slow pace, as demonstrated in the next section. The dependence of the refractive index modulation and the UV dose is detailed in Supplementary Information. The inset in Figure 2d shows the end face of the fabricated single-mode fibre.

**Figure 3:**
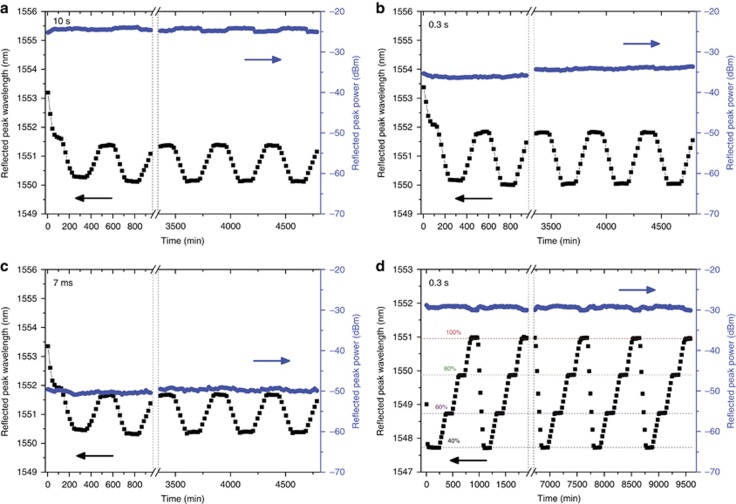
Wavelength and peak power fluctuations during temperature and humidity cycling. The temperature profile was set between 20 and 50 °C. Each cycle consists of 2 h for stabilization at 20 and 50 °C and 2 h for the increase and decrease between the two temperatures, which totalled 8 h. The results of 10 cycles are recorded for (**a**) 10 s FBG, (**b**) 0.2 s FBG and (**c**) 7 ms FBG. The results for 10 humidity cycles between 40%RH and 100%RH at a constant temperature of 40 °C are shown in **d**. Each cycle consists of 2 h for stabilization at each humidity level and 2 h for the increase and decrease between the stated levels. For all of the graphs, only the first two and last three cycles are plotted, with the dash line representing the missing cycles.

To characterize the stability of the FBGs for biomedical applications, thermal tests were conducted using an environmental chamber (ESPEC, SH-641) with the humidity set at 30%. The FBGs were placed inside the chamber with a temperature profile program for 8 h, in the range of 20–50 °C. Ten cycles of the program were executed. Figure 3a–3c shows the results of the stabilized FBGs inscribed in 10 s, 0.3 s and 7 ms, respectively.

The Bragg peak shifts to a shorter wavelength with a higher temperature, which is consistent with previous findings^30, 32, 33^. The initial shift for all of the FBGs within the first 2 h was due to a decrease in the humidity from that of ambient air to 30%, while the temperature was stabilized at 20 °C. Interestingly, all of the FBGs exhibit clear stability. Furthermore, the FBGs exhibited a good thermal response, and their average sensitivities during the last three cycles were approximately −55 pm °C^−1^ for the 0.3 s FBGs and around −40 pm °C^−1^ for the 10 s and 7 ms FBGs. The difference in the sensitivity or temperature coefficient is mainly due to the unintended inhomogeneity of the fibre. Figure 3d shows the results of 10 humidity cycles performed at a temperature of 40 °C. The test protocol was similar to the protocol used for temperature cycling. Remarkable peak power stability is demonstrated, and a sensitivity of 54.2 pm %RH^−1^±0.14%RH was recorded. These test results demonstrated the excellent thermal and humidity stability of the fabricated FBGs, qualifying it for biomedical applications and potentially for *in vivo* sensing.

### Multiple vital signs monitoring using FBG sensors

Silica glass and polymer FBG sensor heads were attached to the subject’s brachial artery location using medical tape (Supplementary Fig. S4). The system consisted of an interrogator (Micron Optics, SM130) with a sampling rate set at 1 kHz, which permitted collection of the peak wavelength shift. As the POF-FBG reflected signal was low, an optical gain of 20 dB was set on the Micron Optics software, allowing easier automatic peak detection. The data were recorded using a Labview program, and each test lasted for 1 min. The heartbeat measurements were compared with a medical device (Nellcor, N20-PA handled pulse oximeter) based on photophlethysmography, with an accuracy of±3 bpm. The medical device is composed of an infrared light source and a detector. The heartbeat is responsible for a change in the blood volume of the finger. As the light is partially absorbed by the blood, recording the reflected light gives the value of the heartbeat.

### Heartbeat measurements at the brachial artery location

**Figure 4:**
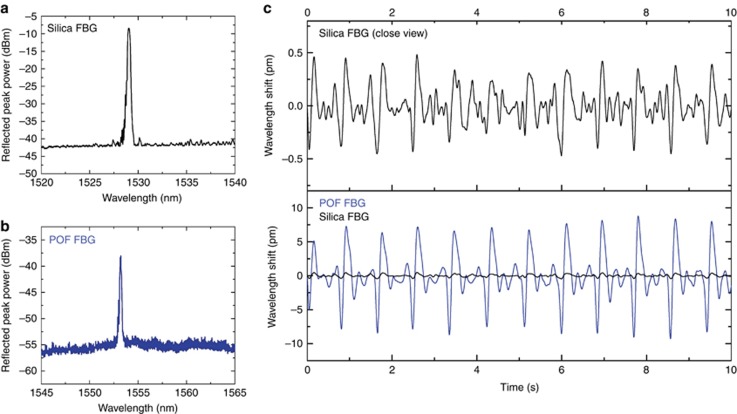
FBG waveforms generated from heartbeat monitoring. (**a**) Reflection spectrum of the silica FBG used in the experiment. (**b**) Reflection spectrum of the 7 ms polymer FBG (stabilized for a few months after UV exposure) used in the experiment. (**c**) Filtered reflection peak wavelength shift of the silica (top) and polymer FBG (bottom).

The reflection spectrum for a typical silica FBG is shown in Figure 4a, and the reflection spectrum of a 7-ms POF grating that was used in the experiment is shown in Figure 4b. The POF-FBG used was manufactured 10 months earlier and exhibited reflected peak power growth within this time. A demonstration was conducted for monitoring the pulse rate using the two FBG sensors.

A comparison of the experimental results obtained with FBGs inscribed in the two materials shows that the polymer sensor has a much larger wavelength shift (approximately 15 pm) than that of the silica sensor (approximately 0.8 pm), as shown in Figure 4c. This finding suggests that there is a sensitivity improvement of approximately 20 times by using a polymer FBG instead of a conventional silica FBG^14^. Experimental results obtained with the POF-FBG match those measured using a medical device, which demonstrates the quality and accuracy of the measurements. The reliability of the POF bio-sensor was further investigated and is presented in Supplementary Fig. S5 in Supplementary Information. The filtered pulse shape acquired by the POF grating^34^ can be used to estimate the blood pressure, as demonstrated by Katsuragawa and Ishizawa^14^. It is important to note that the measured wavelength shift of <2 pm obtained with the silica FBG sensor is similar to that reported by Katsuragawa and Ishizawa^14^, who used a much higher-resolution FBG interrogator.

### Heartbeat and respiration record on chest

**Figure 5:**
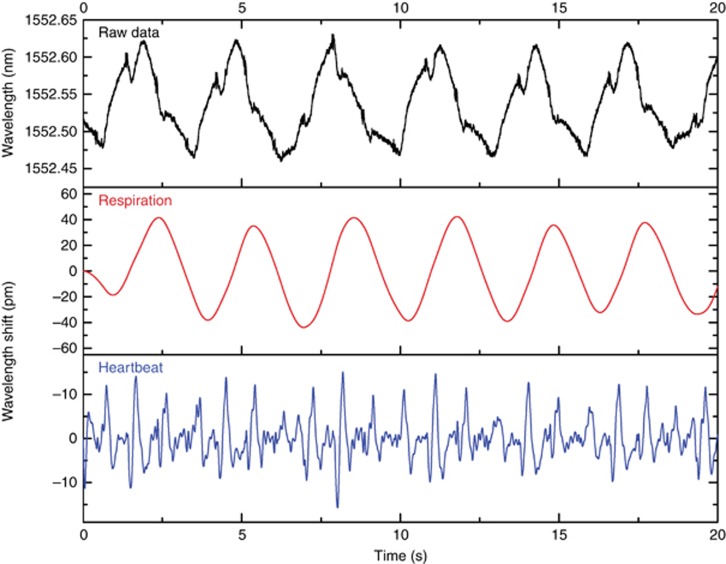
Waveforms for simultaneous respiration and heartbeat monitoring. (Top) Raw data for the reflection wavelength peaks. Waveforms generated after filtering the raw data to monitor the (middle) respiration and (bottom) heartbeat.

Another study was conducted for multiple human vital sign monitoring using the 7 ms polymer FBG. For this demonstration, a single FBG was attached to the subject’s chest. Figure 5 shows the raw data for the detection of the reflection peak wavelengths; the extracted waveforms generated from respiration and heartbeats are separately shown in the figure. The wavelength shift obtained for the filtered respiration signal is approximately 80 pm, whereas it was approximately 17 pm for the filtered heartbeat signal. These results are more accurate and reliable than other results using POF macro-bending sensors^15, 16^ and optical time-domain reflectometer-based sensors^17^ without requiring special preparation nor expensive equipment. Further experiments were conducted together with a comparison with silica FBG, and the findings are presented in Supplementary Information (Supplementary Fig. S6a–S6d). It is demonstrated that POF-FBG exhibit wavelength shifts that are at least 30 times larger than those of silica FBG for both respiratory function and heartbeat monitoring. Note that silica FBG embedded in textiles demonstrated a sensitivity of 150 pm for respiration monitoring and approximately 8 pm for cardiac activity^35^. However, the FBG was embedded in a polyvinyl chloride foil substrate that enabled a large sensitive area, permitting higher sensitivity.

### UV-induced changes in PMMA and DPDS

**Figure 6:**
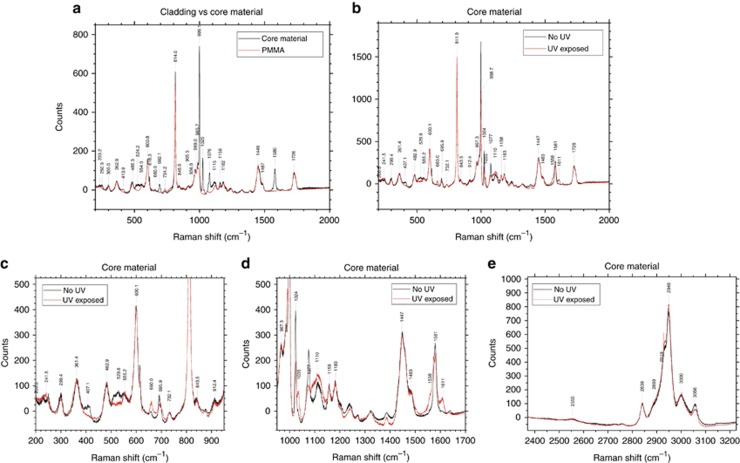
Raman spectroscopy of fibre materials. Panel (**a**) shows the comparison between the core and the cladding materials, while panel (**b**) shows the effect of UV exposure on the core. Closer views of **b** are shown in **c** and **d**, while **e** shows the longer Raman shift part of the spectrum.

Raman spectroscopy was conducted on embedded POFs and core preforms to study the molecular changes caused by the FBG writing process. The signal from the core of a fibre was mainly dominated by the PMMA, and thus the core material preforms were investigated to study the effect of UV on the PMMA–DPDS mixture. Figure 6a shows the Raman spectra obtained when the core materials were tested and compared with the cladding. Peak identification was conducted using Raman spectra found in the literature for DPDS, similar phenyl-based molecules and PMMA^36, 37, 38, 39^. These spectra are very similar, unsurprisingly, because the core is mainly the same material as the cladding, with the differences arising from the DPDS.

The first difference between the spectra is a small peak at 250.9 cm^−1^, which is attributed to the bending vibration of a C-S-S group (δ(C-S-S)). A second clear difference is seen at 413.9 cm^−1^, a frequency that has contributions from both a phenyl mode (Φ_7a_) and a stretching S-S (υ(S-S)) bond. The next difference is seen at 524.2 cm^−1^, which is attributed to υ(S-S). The sharp peak at 616.3 cm^−1^, attributed to a phenyl mode (Φ_6b_), is not seen in the cladding spectra. A small peak is observed in the core spectra at 660.0 cm^−1^ (Figure 6a), which is attributed to the C-S bond. The peak at 692.1 cm^−1^ has contributions from both a phenyl mode (Φ_6b_) and the stretching of the C-S-S bond (υ(C-S-S)). These differences in the peaks are caused by phenyl, C-S-S, C-S or S-S vibrations, which confirms the successful detection of DPDS in the core material of the fibre.

Large differences seen at 999.1, 1025, 1076 and 1580 cm^−1^ are all attributed to various phenyl vibrations, although the 999.1 cm^−1^ has contributions from the O-CH_3_ rock, and the 1076 cm^−1^ also has a contribution from υ(C-S-S) vibrations. Owing to its many vibrational modes and resonances, the phenyl signal is stronger than the contribution from the sulphur-related bonds.

Comparison between spectra taken from the core before and after the fibre is exposed to UV, shown in Figure 6b, reveals changes to the bonding of these molecules. These are shown in Figure 6b, with closer views in Figure 6c and 6d.

After UV exposure, the peak at 250.9 cm^−1^ has shifted to a lower wavenumber and merged with the peak at 233.2 cm^−1^. The next change is seen at 407.1 cm^−1^, with the Φ_7a_/υ(S-S) peak no longer present. There is also a decrease in the intensity of the υ(S-S) peak at 529.8 cm^−1^. A small increase in the peak at 660.0 cm^−1^ is seen, while a small decrease in the adjacent peak at 695.9 cm^−1^ is attributed to a combination of Φ_6b_ and υ(C-S-S). The phenyl signal is seen to decrease, sometimes significantly, at 616, 998.7, 1024 and 1077 cm^−1^. A peak at 1035 cm^−1^ is seen to increase. Peaks that split or develop shoulders are also of interest. The 998.7 cm^−1^ peak, associated with Φ_12_ and the O-CH_3_ rock, decreases after UV exposure, while a small shoulder develops at 991.3 cm^−1^. A similar change is seen at the 1580 cm^−1^ peak (Φ_8a_), with a splitting into two peaks that are very close in wavenumber. Two further shoulders have also developed at 1558 and 1611 cm^−1^. This shows a significant change to the phenyl ring Φ_8a_ vibrations after UV exposure. Some small changes in the bonds that involve hydrogen are shown in Figure 6e, although it should be noted that the -S-H bond vibration at 2555 cm^−1^ is not seen to change.

These results can be mainly split between changes in the carbon–sulphur bonding and changes to the phenyl rings. Both can provide information about what occurs inside a fibre after UV light exposure and will be discussed further in the next sections.

### Chemical modelling of a fibre system

**Figure 7:**
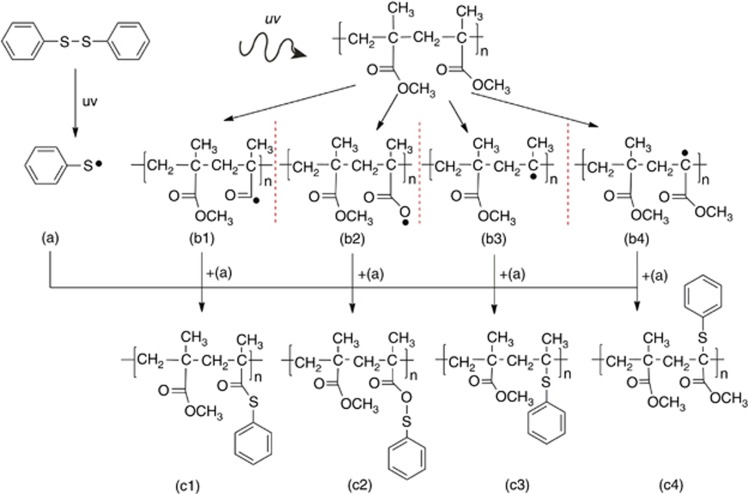
Chemical reactions considered in the energy calculations. Upon UV exposure, we assume that DPDS is cleaved into two sulphenyl radicals (**a**) while the PMMA chain (here modelled by only two monomers linked together) undergoes various photodegradation reactions, four possibilities of which are considered here (**b1**–**b4**). These are the removal of the OCH_3_ group from the side chain (**b1**), removal of the CH_3_ group on the side chain (**b2**), complete removal of the side chain (**b3**) and removal of the CH_3_ group opposite the side chain (**b4**).

Electronic structure calculations were used to further investigate the effect of UV exposure on the POFs. The simulation parameters are given in the Materials and Methods section. Various possible reaction pathways were considered to determine which was the most thermodynamically favourable. These are outlined in Figure 7.

It was assumed that the UV light causes homolysis of the DPDS molecule, splitting it into two sulphenyl radicals, while various possible photodegradation effects were considered on a ‘mock PMMA’ chain that consists of two monomer units. These photodegradation effects were the removal of the OCH_3_ group from the side chain, removal of the CH_3_ group on the side chain, complete removal of the side chain and removal of the CH_3_ group from the main chain. These options were considered because damage to the side chains of PMMA is known to occur after UV exposure^40, 41^. It was found that the attachment of the sulphenyl radical to the side chain after OCH_3_ removal is the most energetically favourable, followed by the attachment to the main chain after complete side chain removal, as shown by the change in free energies given in Table 1. The other attachments to various parts of the PMMA molecule were also found to be more energetically favourable than recombining with another sulphenyl radical.

### The photosensitivity of the POF and growth dynamics of the FBGs

The mechanism that leads to photoinduced index changes in PMMA optical fibre under UV irradiation is complicated and still requires further investigation. The wavelength of 325 nm is used for the majority of the work^42^ on FBG inscription in POFs because PMMA starts to degrade upon UV irradiation below approximately 300 nm^6^, and 325 nm is one of the main emission lines of a HeCd laser. FBG formation in POFs continues for a long time (ranging from a few weeks to a few months) after the initial UV exposure.

The S-S bond of DPDS can be broken either by heat or under UV irradiation^43, 44, 45^ because it has a low bond dissociation energy (214.2 kJ mol^−1^)^46^ compared with other core dopants such as diphenyl sulphide (327.6 kJ mol^−1^). Thermal homolysis occurs at ~300 °C^47^, which is higher than any temperatures achieved during fibre processing.

Under UV irradiation, homolysis of the DPDS occurs, and two sulphenyl radicals are created^44^. Disulphide bonds have been shown to break in ~2 μs after UV absorption in a protein system^48^. Therefore, with two phenyl groups attached by a disulphide bond, DPDS will undergo photodissociation on the order of microseconds, and the sulphenyl radicals can attach to photodegraded sites on the PMMA chain through the sulphur atom, which is supported by decreases in the Raman peaks associated with S-S bonds (407.1 cm^−1^, 529.8 cm^−1^), C-S-S bonds (695.9 cm^−1^) and a slight increase in C-S bonds (660.0 cm^−1^) (Figure 6b and 6c). DPDS dissociation would also explain the slight increase in the C-S bond observed at 660.0 cm^−1^ after UV exposure (shown in Figure 6c) because breaking a DPDS molecule into two can potentially generate twice as many C-S bonds if the two parts of the molecule join onto a PMMA chain. The negligible change in the υ(-S-H) bond vibration seen at 2555 cm^−1^ (shown in Figure 6e) shows that the cleaved molecule rarely pairs up with any hydrogen radicals that might have been removed from the PMMA chain or elsewhere.

The stability of the phenyl rings^49^ means that the rings are relatively unharmed by the UV exposure^50^. The vibrational modes of the phenyl ring are observed to change because the DPDS molecule splits. Although some peaks are reduced, a few are strengthened after UV exposure, while others have split, broadened or developed shoulders. These changes suggest that the phenyl rings are mainly still intact but their attachments are now different. The peak splitting is especially indicative of there being several different possible configurations after the UV exposure. The development of the small shoulder at 991.3 cm^−1^ could also be due to full or partial scission of the side chain^51^, although they would be difficult to isolate from changes in the phenyl signal at the same wavenumbers. The addition of aromatic rings and sulphur atoms is known to increase the refractive index of polymers^52^.

Simulation results show that the most likely routes are the cleaving of the OCH_3_ from the side chain or total side chain scission, followed by replacement with the sulphenyl radical through the sulphur atom. This procedure agrees with the changes seen via Raman spectroscopy. These changes cause the rapid positive change in the refractive index achieved during the Bragg grating inscription when using DPDS as a dopant, which permits the grating to appear immediately after UV irradiation (Figure 2a). Shorter inscription times would be possible using a higher power 325 nm laser and a faster optical beam shutter.

When the UV fluence is low (<65.8 mJ cm^−2^), not many bonding sites are available on damaged PMMA chains (Norrish type I photochemical reaction^53^), and the final configuration is mostly uniform, giving well-defined reflection peaks in Figure 2a–2c. FBGs inscribed with higher UV energy fluences (>65.8 mJ cm^−2^) show evidence of the effect of Norrish type II photochemical reaction^53, 54^, giving rise to more numerous final molecular configurations^53^, which creates irregularities along the irradiated regions of the fibre and thus the grating. This complexity is likely the cause of the higher noise seen in the FGBs written with longer exposure times, which is seen in Figure 2a–2c. The FBGs exhibited very good thermal stability (Figure 3), which is a well-established characteristic of vulcanization^55^. The exact cause of the growth of the reflected peak power of the gratings (Figure 2d) over several weeks requires further investigation, although these results suggest that further molecular rearrangement occurs over that time.

## Conclusions

The discovery of DPDS as a single material for increasing both the refractive index of POFs and their photosensitivity is announced in this paper. This approach allows the rapid writing of FBGs. FBG inscription in just 7 ms is achieved, which is more than two orders of magnitude faster than the previously reported result of writing FBGs in POFs at the 325 nm wavelength. In this article, a demonstration of multiple vital signs monitoring using the 7 ms polymer FBG was conducted. The results show approximately 20 times improvement in the sensitivity for heartbeat measurements at the brachial artery location compared with silica-based FBG and at least 30 times improvement for both respiratory function and heartbeat monitoring at the chest location. An explanation of the photosensitivity, growth dynamics and thermal stability of FBGs inscribed in DPDS core-doped fibres is presented. These findings enable high-quality gratings in POFs to be produced during the fibre-drawing process—a significant step towards developing low-cost, single-use polymer fibre-sensing systems for biomedical applications. This result should also be of interest to the wider precision sensor industry, while further applications in Internet of Things and automation can also be envisioned.

## Figures and Tables

**Table 1:** Free energies of UV-induced reactions

Reaction	Note	*ΔG*	*ΔH*	*ΔE*+ZPE
(a)+(a)→DPDS	Recombination of sulphenyl radicals	−22.25	−34.23	−34.40
(a)+(b1)→(c1)	OCH_3_ removed from side chain	−37.43	−51.62	−51.26
(a)+(b2)→(c2)	CH_3_ removed from side chain	−24.65	−36.47	−36.55
(a)+(b3)→(c3)	Side chain removed	−30.43	−46.08	−45.34
(a)+(b4)→(c4)	CH_3_ removed from main chain	−23.34	−37.07	−36.90

The relative energy values (in kcal mol^−1^) of Δ*G*, Δ*H* and Δ*E*+ZPE calculated at the B3LYP/6–311++G(d,p) level of theory.
