# Supplementary material for: Ultra-fast polymer optical fibre Bragg grating inscription for medical devices
Source: Light Sci Appl. 2018 Mar 23;7:17161–. doi: 10.1038/lsa.2017.161 (PMC6060050; doi:10.1038/lsa.2017.161)
Supplement: Supplementary Information [file lsa2017161x1.docx]

SUPPLEMENTARY INFORMATION

**Ultra-fast polymer optical fibre Bragg grating inscription for medical devices**

Julien Bonefacino^1*^, Hwa-Yaw Tam^1*^, Tom S Glen^1^, Xin Cheng^1^, Chi-Fung Jeff Pun^1^, Jian Wang^2^, Po-Heng Lee^2^, Ming-Leung Vincent Tse^1^, and Steven T Boles^1^

^1^ Photonics Research Centre, Department of Electrical Engineering, The Hong Kong Polytechnic University, Hung Hom, Kowloon, Hong Kong SAR.

^2^ Division of Urban Environment, Department of Civil and Environmental Engineering, The Hong Kong Polytechnic University, Hung Hom, Kowloon, Hong Kong SAR.

*email: [julien.bonefacino@gmail.com](mailto:julien.bonefacino@gmail.com); [hwa-yaw.tam@polyu.edu.hk](mailto:hwa-yaw.tam@polyu.edu.hk)

# Preform fabrication using the pull-through method

Two steps were employed to fabricate our polymer preform, namely a pre-polymerisation stage and a polymerisation stage. The pre-polymerisation process took place in a sealed glove box while the polymerisation process was carried out in an oven. Chemicals were weighed using an electronic balance (Starorius BSA124S) to get the required ratio between monomer methyl methacrylate, initiator and chain transfer for fabrication of the cladding preform. All the chemicals were mixed in a beaker and placed in a glove box filled with argon gas at a pressure of 2.5 bars to the standard of ISO 5 (particles of 0.5 µm < 3250 counts in the ISO 14644-1 cleanroom standards). In order to prevent explosive polymerisation from occurring, the solution was stirred and heated in an oil bath at temperature of ~80 °C for 20 minutes inside the argon filled environment. A particle counter (Met One Instruments GT-321) was used to record particles with sizes of 0.3 µm, 0.5 µm, 1 µm, 2 µm and 5 µm. Temperature and humidity were recorded with a commercial detector. This procedure allowed us to fabricate preforms with fewer dust particles and lower humidity. After the pre-polymerisation process, the viscous solution was poured into a 20 mm test tube, in which a 0.8 mm Teflon string was fixed along the central axis. The ensemble was then placed in an oven (MEMMERT 500) with a specific temperature profile, increasing from 70 °C to 110 °C over a period of 96 hours, to complete the polymerisation process. The string was then pulled out of the preform, leaving the cladding-like part with a hole running along the central axis. Separately, the core mixture was prepared in the same way and poured into a 13 mm test tube. After curing in the oven, the core preform was drawn into a 0.8 mm rod using a custom-made POF drawing tower. The rod was tightly inserted into the 20:0.8 mm cladding preform. The resultant preform was then drawn into the final fibre. The temperature of the infra-red heater was maintained at around 200 °C, and the draw tension was about 0.05 N. The final fibre diameter was 120 µm with 3 % tolerance.

# FBG fabrication process and alignment with silica fibre

For alignment purposes only, a mock grating was written near the far end of a 5 cm long POF. This was done with spectral monitoring and shows the robustness of the inscription system and the reproducibility of grating fabrication. Next, the alignment of the cores of the POF and silica (SMF) fibres was carried out by maximising the reflection signal using a 3 axis micro-positioning stage. The SMF was cleaved at an angle greater than 8 degrees at the joint to annihilate the Fresnel reflection. Next, the joint of the two fibres was glued using a UV curable epoxy. Thus the epoxy was acting as an adhesive as well as an index matching medium (*n* = 1.50). After the epoxy was cured, the mock grating was removed, leaving a 3 cm long POF connected to the SMF. This process was also a good way to ensure the quality of a particular piece of fibre. Finally, the polymer FBG under investigation was written far from the joint. For vital sign monitoring, the gluing point was protected with a 1 cm long cylindrical tube in which a UV curable glue was inserted offering good protection of the gluing point.

# DPDS intrinsic loss

The loss introduced by DPDS in PMMA was measured in un-cladded 120 µm diameter PMMA fibres doped with 0 %wt (pure PMMA), 2 %wt (1 %mol), 4 %wt (2 % mol) and 8 %wt (4 %mol) of DPDS.

The attenuation measurements were made using the cut-back method. A 650 nm laser diodes module and a 870 nm SLD (Exalos) were used for the measurements. The power was measured using a power meter (Thorlabs, PM320E) coupled with an integrating cavity sphere detector (Thorlabs, S142C). Fibres with length of 50 cm were taken at different positions on a spool and cut 10 times repeatedly by 1 cm. For each fibre five tests were made. Figure S1a and S1b show results obtained at 650 nm and 870 nm, respectively.


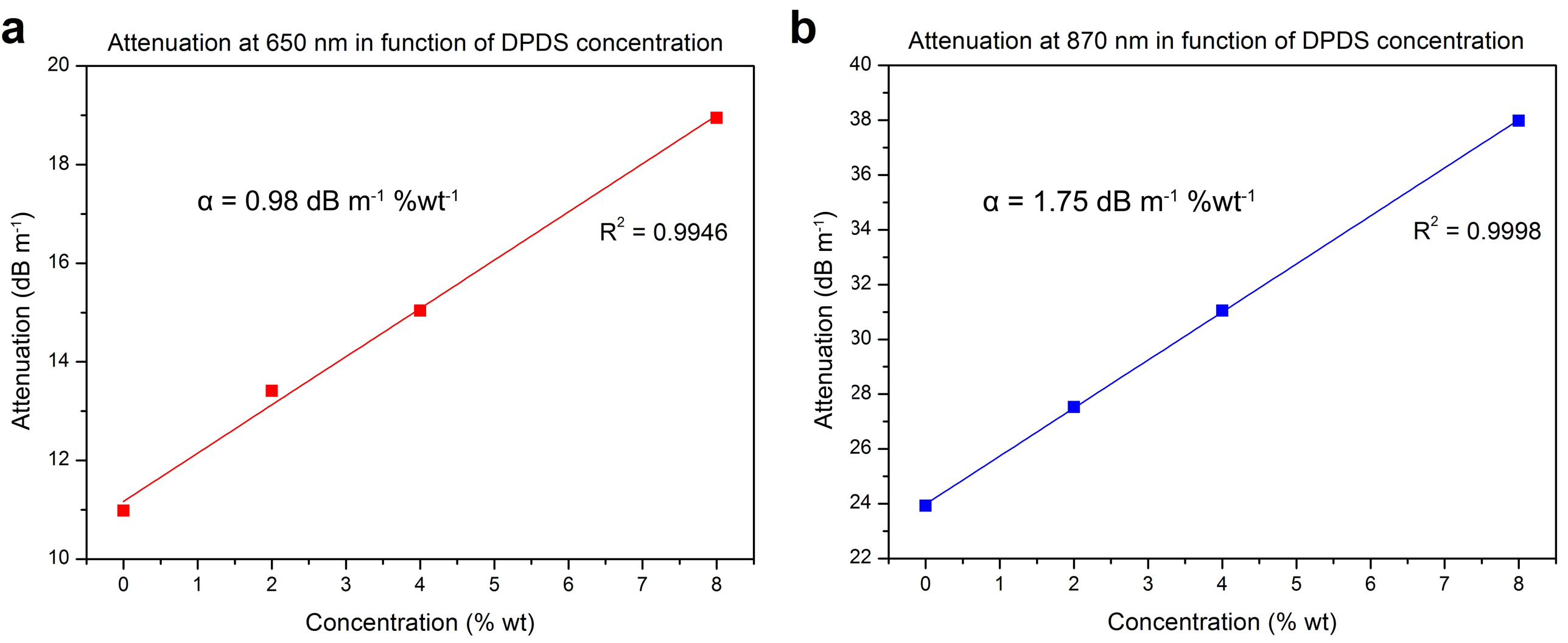


**Figure S1** Attenuation measurement in un-cladded DPDS-doped PMMA fibres. **(a)** measured at 650 nm, **(b)** measured at 870 nm.

# Reproducibility of grating manufacturing in POFs

The Figure S2 below shows the reproducibility of grating fabrication in DPDS core doped POF for FBGs fabricated within 0.3 s and 7 ms.


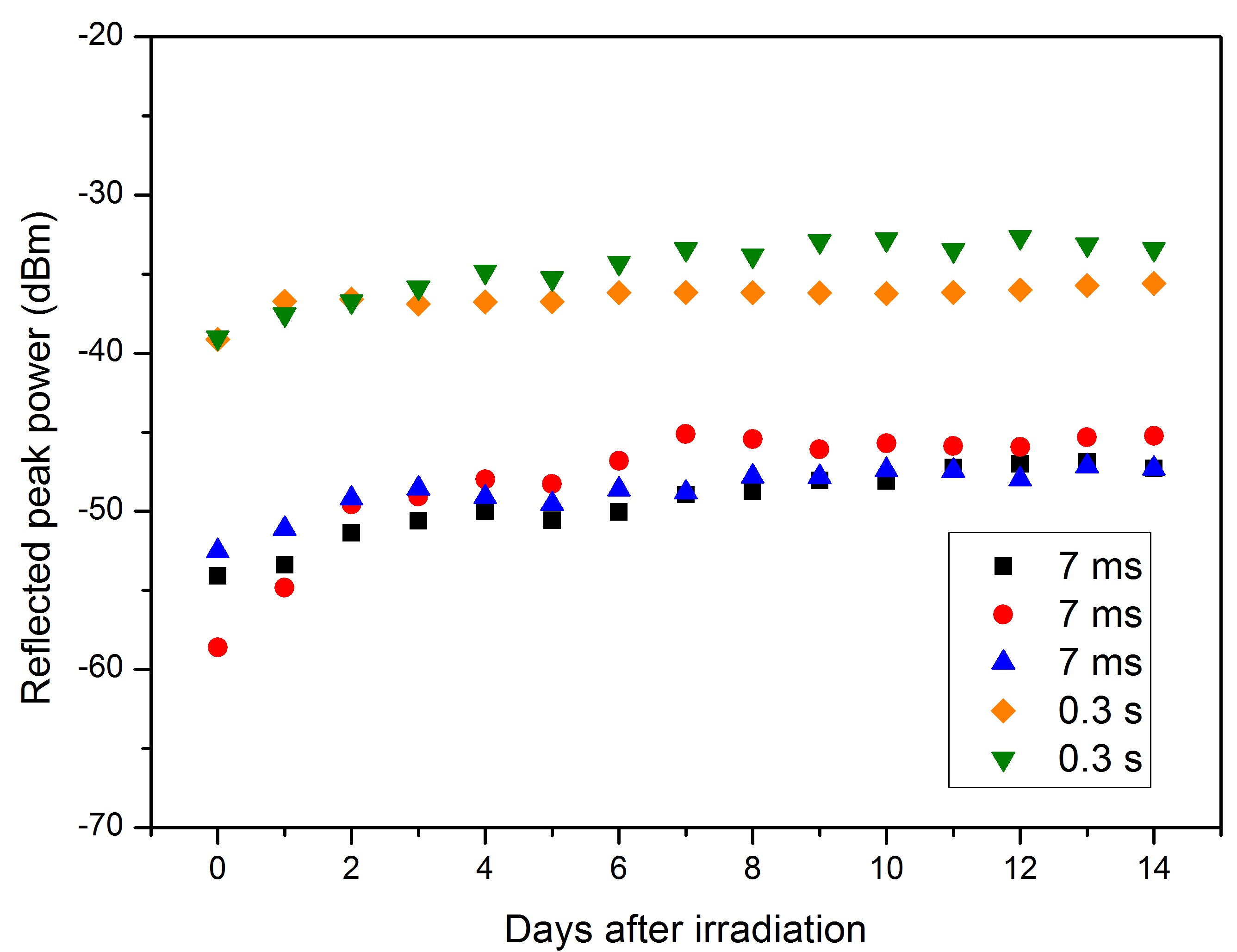


**Figure S2** Repeatability of FBG fabrication for gratings written using 0.3 s irradiation time and 7 ms irradiation time.

# Refractive index modulation upon UV irradiation





**Figure S3** Refractive index measurements of DPDS core doped single mode polymer optical fibres. **(a)** refractive index modulation for fibre irradiated 28 ms, **(b)** refractive index modulation for fibre irradiated 0.8 s, **(c)** refractive index modulation for fibre irradiated 4 s. **(d)** refractive index modulation in function of irradiation time.

The refractive-index profile of the DPDS-doped POFs was measured by Interfiber Analysis, LLC (http://interfiberanalysis.com) based in Sharon, Massachusetts, USA. All the samples sent for measurement were 20 cm long and 1 cm long region around the middle of the fibres irradiated with 325 nm beam. Figure S3a, S3b, and S3c show the results of three samples irradiated with 325 nm light directly (without phase-mask) for 28 ms (to simulate 7 ms of UV irradiation through the phase-mask), 0.8 s, and 4 s, respectively.

# Repeatability of heartbeat measurement at the brachial artery location

Figure S4 below shows the set-up of both polymer and silica FBGs on the brachial artery location.


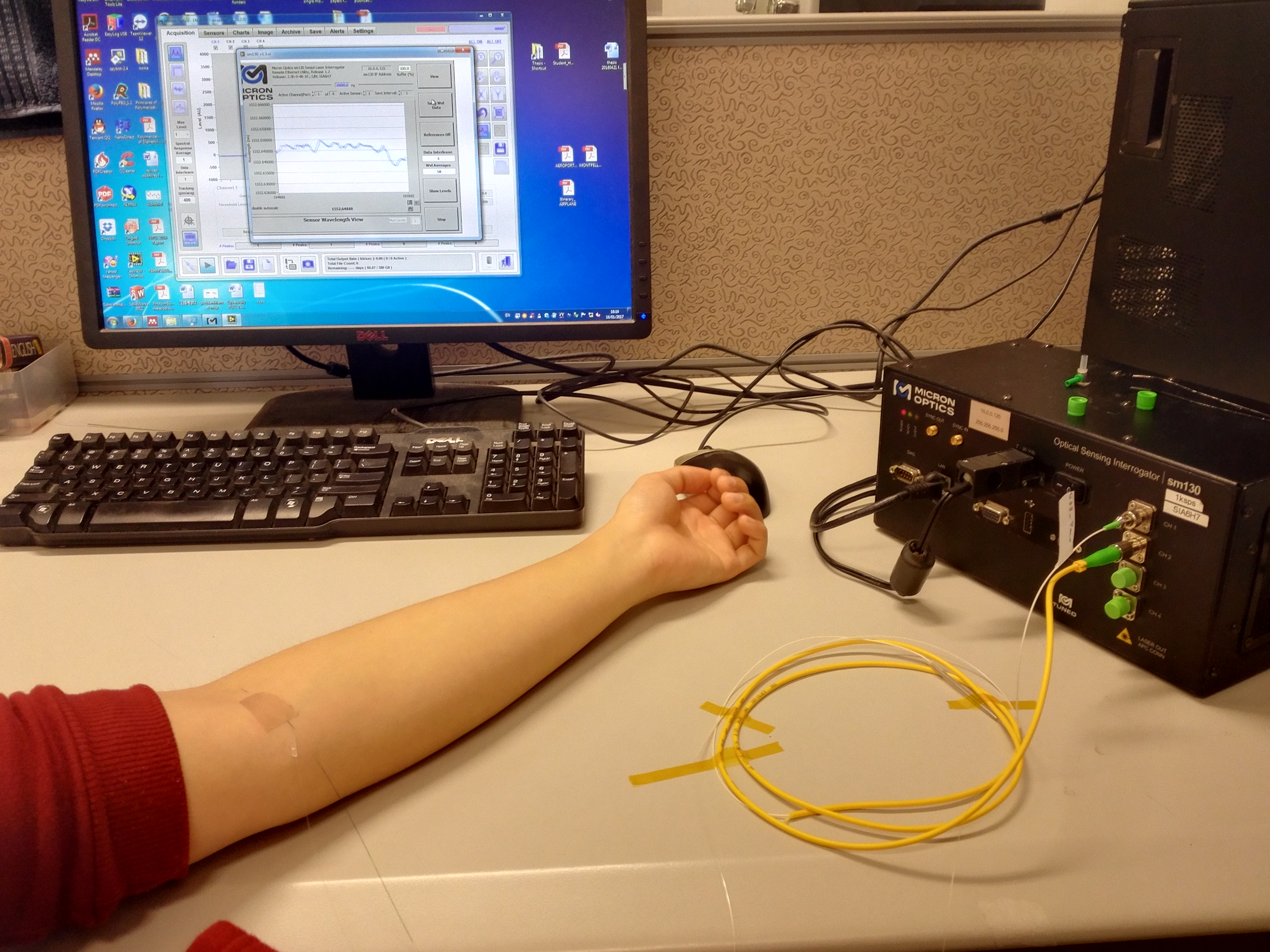


**Figure S4** Polymer fibre optics pulse monitoring system. Silica glass FBG and polymer FBG were attached to a subject’s brachial artery arm location using medical band-aid tape to measure pulse wave shape.

To further confirm the quality and repeatability of the measurements performed at the brachial artery position, four more tests were performed on male subjects with ages ranging from 30 to 40 years old. The test conditions and filtering process were the same as described in the manuscript. Figure S5 below shows the filtered data obtained.

Very good results were obtained on the four subjects and the heartbeat recorded within 1 min can be easily extracted by applying a threshold to the filtered data. The heartbeats were respectively 64 bpm, 69 bpm, 71 bpm, and 71 bpm, which perfectly fit the value given by the medical device control. Excellent results were obtained on subject A, which explain the difference scale used on Figure S5. The average wavelength shift recorded was of 25 pm for subject A and around 15 pm for the three remaining subjects.


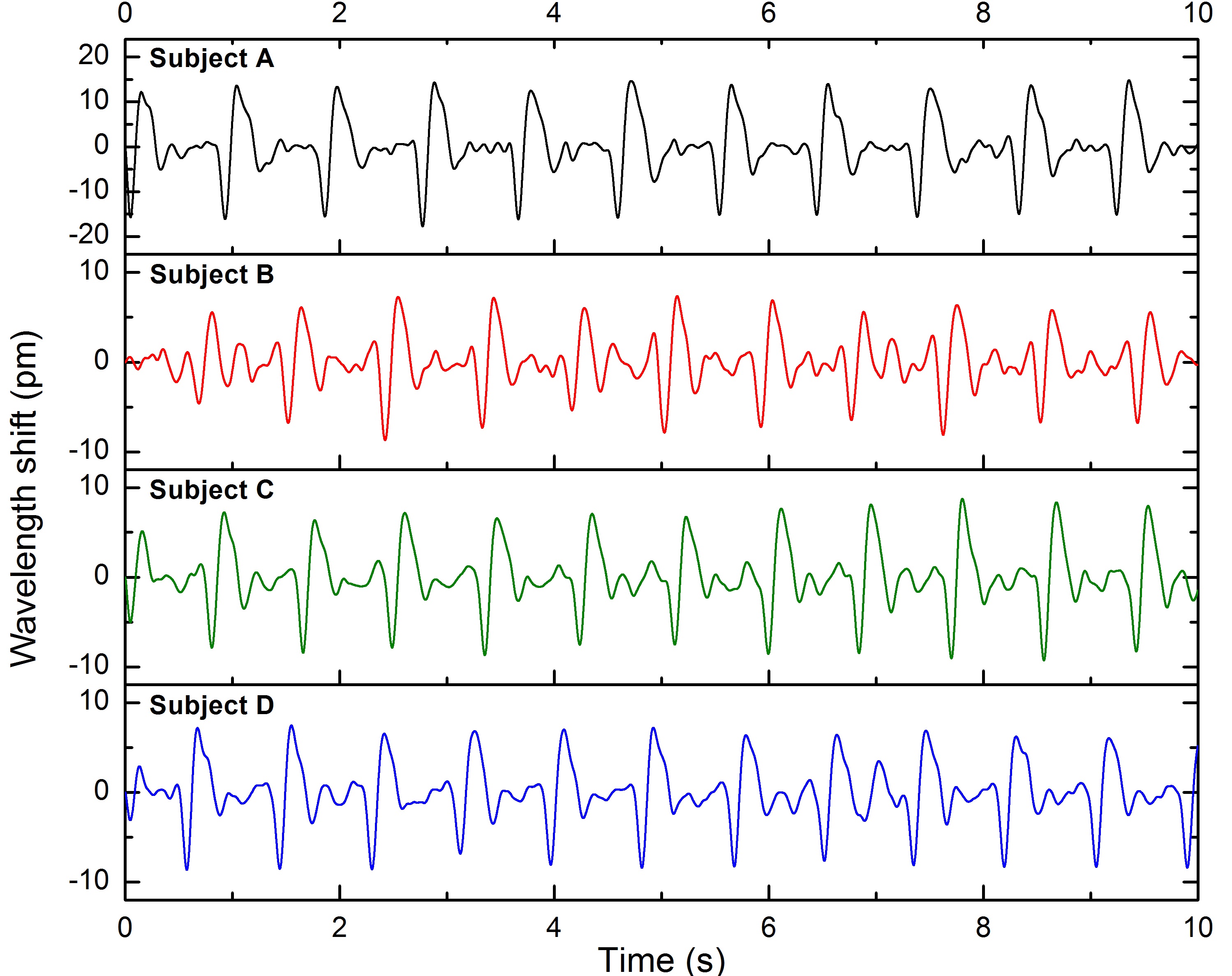


**Figure S5** Heartbeat measurements performed at the brachial artery location on 4 male subjects with age ranging from 30 to 40 years old.

# Respiration and heartbeat measurements at the heart location

Tests using silica FBG and 7-ms POF FBG were performed at the heart location. The subject was asked to start holding their breath after 30 s. Two tests were performed in which the subject was asked to hold their breath after exhaling air (Figure S6a and S6b), and after inhaling air (Figure S6c and S6d). These two configurations were interesting in the sense that the thoracic cage was either relaxed or under tension. The record of Figure S6 clearly shows that POF-FBG based technology are much more sensitive than silica FBG. Indeed, in both tests, the raw data for the 7-ms POF-FBG are sufficient to notice the respiratory function and the moment at which the subject has stop breathing. Furthermore, the average peak to peak wavelength shift induced by respiration during the first test was over 150 pm for POF (Figure S6b) whereas it was only 2 pm for silica grating (Figure S6a). This demonstrates an improvement of 75 times using POF. Furthermore, the heartbeat induced wavelength shift was of 15 pm for polymer fibre and only 0.5 pm for silica fibre. Besides, the filtered heartbeat signal exhibited more noise for silica grating compared to POF-FBG. Interestingly, the wavelength shift recorded for POF-FBG was more than two times larger (Figure S6b) during inhalation. As the breath was held after an exhalation, the sensitivity within the 20 last seconds of experiment was lower.


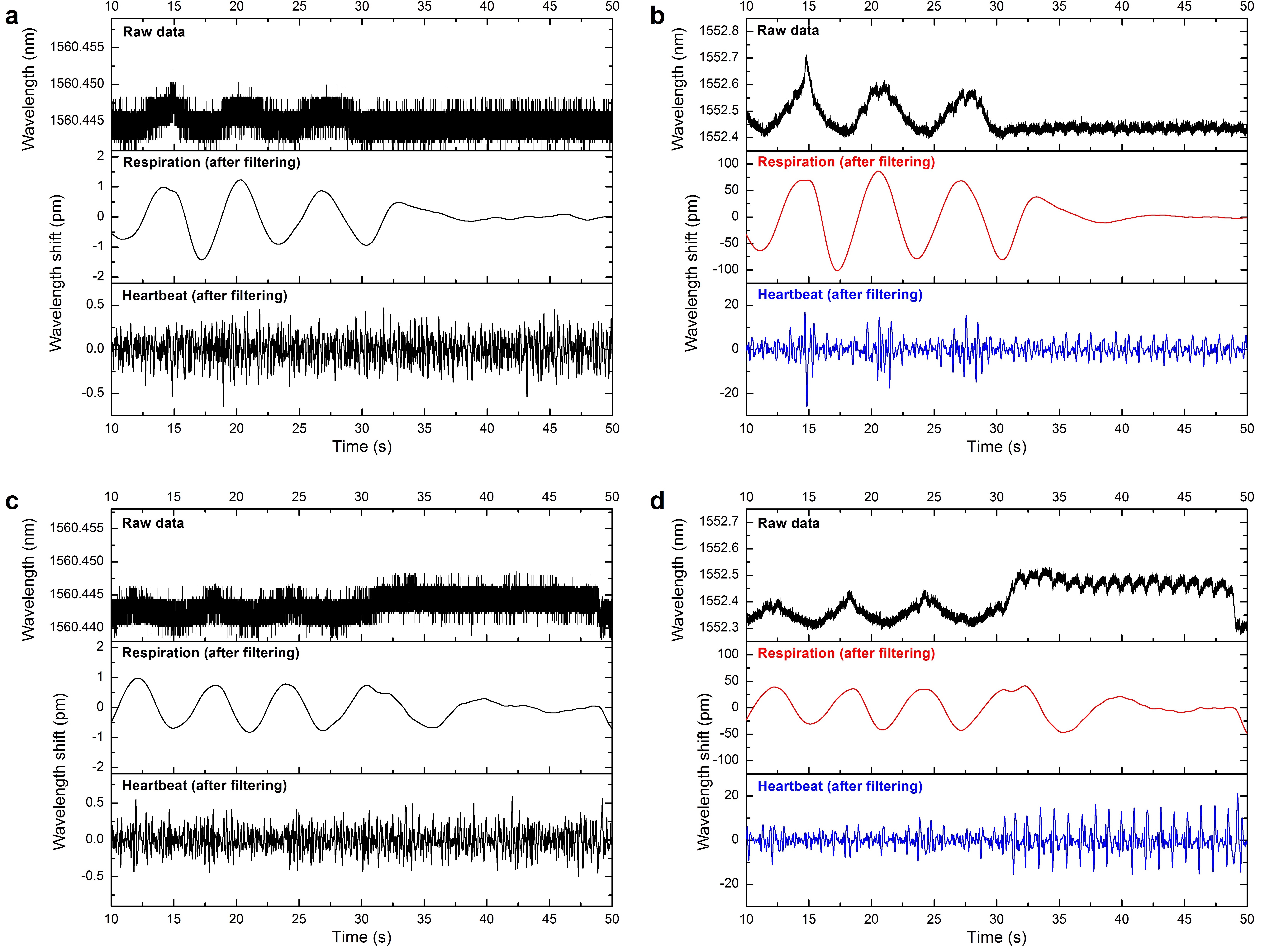


**Figure S6** Respiration and heartbeat measurements performed at heart location. Records obtained using silica and POF-FBG once breath was held after exhalation are presented in **(a)** and **(b)** respectively. Records obtained using silica and POF-FBG once breath was held after inhalation are presented in **(c)** and **(d)** respectively.

Confirmation of those results is demonstrated in Figure S6c and S6d. Silica fibre shows wavelength shifts of 2 pm and 0.5 pm for respiratory function and heartbeat respectively (Figure S6c), whereas it was of 75 pm and 15 pm for POF (Figure S6d). The difference in sensitivity recorded for POF is due to the different location at which the fibres were placed. Once breath is held after an inhalation (Figure S6d), the wavelength shift recorded for heartbeat increased to 25 pm for POF fibre.
